# Quantification of *SLIT-ROBO *transcripts in hepatocellular carcinoma reveals two groups of genes with coordinate expression

**DOI:** 10.1186/1471-2407-8-392

**Published:** 2008-12-29

**Authors:** Mehmet Ender Avci, Ozlen Konu, Tamer Yagci

**Affiliations:** 1 Department of Molecular Biology and Genetics, Bilkent University, Ankara, Turkey

## Abstract

**Background:**

SLIT-ROBO families of proteins mediate axon pathfinding and their expression is not solely confined to nervous system. Aberrant expression of *SLIT-ROBO *genes was repeatedly shown in a wide variety of cancers, yet data about their collective behavior in hepatocellular carcinoma (HCC) is missing. Hence, we quantified *SLIT-ROBO *transcripts in HCC cell lines, and in normal and tumor tissues from liver.

**Methods:**

Expression of *SLIT-ROBO *family members was quantified by real-time qRT-PCR in 14 HCC cell lines, 8 normal and 35 tumor tissues from the liver. ANOVA and Pearson's correlation analyses were performed in R environment, and different clinicopathological subgroups were pairwise compared in Minitab. Gene expression matrices of cell lines and tissues were analyzed by Mantel's association test.

**Results:**

Genewise hierarchical clustering revealed two subgroups with coordinate expression pattern in both the HCC cell lines and tissues: *ROBO1*, *ROBO2*, *SLIT1 *in one cluster, and *ROBO4*, *SLIT2*, *SLIT3 *in the other, respectively. Moreover, *SLIT-ROBO *expression predicted *AFP*-dependent subgrouping of HCC cell lines, but not that of liver tissues. *ROBO1 *and *ROBO2 *were significantly up-regulated, whereas *SLIT3 *was significantly down-regulated in cell lines with high-*AFP *background. When compared to normal liver tissue, *ROBO1 *was found to be significantly overexpressed, while *ROBO4 *was down-regulated in HCC. We also observed that *ROBO1 *and *SLIT2 *differentiated histopathological subgroups of liver tissues depending on both tumor staging and differentiation status. However, *ROBO4 *could discriminate poorly differentiated HCC from other subgroups.

**Conclusion:**

The present study is the first in comprehensive and quantitative evaluation of *SLIT-ROBO *family gene expression in HCC, and suggests that the expression of *SLIT-ROBO *genes is regulated in hepatocarcinogenesis. Our results implicate that *SLIT-ROBO *transcription profile is bi-modular in nature, and that each module shows intrinsic variability. We also provide quantitative evidence for potential use of *ROBO1*, *ROBO4 *and *SLIT2 *for prediction of tumor stage and differentiation status.

## Background

*Drosophila slit *and *roundabout *(*robo*) genes were identified in genetic screens of mutants for embryonic patterning and commissural axon pathfinding defects [1]. Subsequently, it was shown that SLIT acts as a ligand for ROBO receptor, preventing axons from recrossing the central nervous system (CNS) midline, and that this binding is conserved among vertebrates including mammals [2,3]. In mammals, three *SLIT *(*SLIT1-3*) and four *ROBO *(*ROBO1-4*) genes have been described [4,5].

*SLIT *and *ROBO *genes are mainly expressed in the CNS but there are affirmative data that they are also expressed in non-neuronal tissues, such as mouse lung and kidney [6,7]. Binding of SLIT2 to ROBO1 inhibits CXCL12-induced chemotaxis of leukocytes, T cells and monocytes [8-10]. However, ROBO4 expression has been found to be confined to vasculature and Robo4 signaling modulates endothelial cell migration [11].

On the other hand, like other developmental pathways, aberrant expression of the *SLIT-ROBO *genes has been observed in a wide variety of cancers. Mice with targeted homozygous deletion of first Ig domain of *Robo1*/*Dutt1 *died at birth because of abnormal lung development, and few survivors eventually developed epithelial bronchial hyperplasia [6]. In breast carcinoma tissue samples *ROBO1 *was shown to be overexpressed while SLIT2 induced migration of breast cancer cell lines [12]. SLIT2-ROBO1 signaling was involved in angiogenesis by increasing microvessel density and tumor mass in a tumor xenograft model [13]. In the same study, *SLIT2 *exhibited overexpression in tumor cell lines and primary tumors of a variety of tissues. In contrast, *SLIT2 *also was proposed to be a tumor suppressor gene, which was silenced epigenetically in lung, breast, colon cancers and gliomas [14-16]. *SLIT3 *was silenced by promoter hypermethylation in gliomas and colorectal cancers [17]. *SLIT1 *and *SLIT3 *were overexpressed in prostate tumors [18], whereas along with *SLIT2 *they were slightly expressed only in poorly differentiated HCC [19]. CXCL12 was reported to activate the migration of human melanoma and breast cancer cells that express CXCR4, ROBO1 and ROBO2, while SLIT2-ROBO interaction was demonstrated to inhibit chemotaxis, chemoinvasion and adhesion of breast cancer cells [20]. Furthermore, ROBO4 was overexpressed in tumor endothelial cells in comparison to normal adult endothelial cells [21].

Despite the compiling evidence of *SLIT-ROBO *deregulation in various tumors, only few reports with apparent controversies exist with regard to the expression pattern of these genes in hepatocellular carcinoma (HCC). The overexpression of ROBO1 in HCC was recently reported and this receptor was proposed as an HCC marker in humans [19]. In contrast, another study reported that *Robo1 *heterozygous mice developed spontaneous HCC tumors [22]. It was shown by immunohistochemical staining that SLIT2 protein also was present in HCC tumor sections [13]. Moreover, karyotyping analyses of HCC do not reveal any chromosomal gains or losses associated with *SLIT-ROBO *genes [23,24]. Therefore, in this study, we quantified *SLIT1*, *SLIT2*, *SLIT3*, *ROBO1*, *ROBO2 *and *ROBO4 *transcripts in HCC cell lines and tissues. We observed that *SLIT-ROBO *genes could be partitioned into two main clusters based on their expression in either the HCC cell lines or tissues. *SLIT-ROBO *expression also clustered the HCC cell lines in two groups according to their *AFP *expression pattern. In liver tissues, differential expression of *ROBO1*, *ROBO4 *and *SLIT2 *was found to be associated with clinicopathological parameters such as tumor staging and differentiation. Herein, we describe a comprehensive *SLIT-ROBO *expression signature in HCC.

## Methods

### HCC Cell Lines and Tissues

13 hepatoma and 1 hepatoblastoma (HepG2) cell lines were included in the study and cultured as previously described [25]. Focus, Hep40, Hep3B, Hep3B-TR, HepG2, HUH7, Mahlavu, PLC/PRF/5, SK Hep1 cells were cultured in low-glucose DMEM supplemented with 10% FBS, 100 U/ml Penicillin-Streptomycin, and 0.1 mM non-essential amino acids (HyClone, Utah, USA). SNU387, SNU398, SNU423, SNU449, SNU475 cells were cultured in RPMI 1640 medium supplemented with 10% FBS, 100 U/ml Penicillin-Streptomycin, 0.1 mM non-essential amino acids (HyClone, Utah, USA). TissueScan Liver Cancer Tissue qPCR Arrays, each containing 40 liver tumor and 8 tumor-adjacent normal tissue cDNAs, were purchased from Origene Technologies, (Rockville, MD, USA). 5 non-HCC tumor tissues consisting of 3 cholangiocarcinomas, 1 nodular hyperplasia and 1 liver adenoma were excluded from the present study. Clinicopathological characteristics of the tissues were presented in Additional file 1.

### Primers

PCR primers for human *SLITs*, *ROBO1 *and *ROBO2 *were previously described [18]. Human *ROBO3*, *ROBO4 *and *AFP (alpha-fetoprotein) *primers were designed using Primer3 and targeting exon-exon junctions in order to prevent amplification of possible contaminating genomic DNA [26]. Primer sequences were as follows: *ROBO3 *forward 5'-CAGTGTCCGATGGAAGAAGG-3' and reverse 5'-GTCCATCTCCTGCACATTGG-3', *ROBO4 *forward 5'-GACACTTGGCGTTCCACCTC-3' and reverse 5'-AGAGCAAGGAGCGACGACAG-3', *AFP *forward 5'-AAATGCGTTTCTCGTTGCTT-3' and reverse 5'-CCAACACCAGGGTTTACTGG-3'. Primer pair for the housekeeping gene *GAPDH (glyceraldehyde-3-phosphate dehydrogenase) *was described before [27]. *ACTB *(beta-actin) primer pair was supplemented in TissueScan Liver Cancer Tissue qPCR Array 1 (Origene Technologies, Rockville, MD, USA).

### RNA Isolation and cDNA Synthesis

Cell lines were grown to confluency in 100 mm dishes. Total RNA was extracted using RNeasy Mini Kit (Qiagen, Hilden, Germany) according to manufacturer's instructions. cDNA was synthesized with random hexamers from 1 μg of total RNA using DyNAmo™ cDNA Synthesis Kit (Finnzymes, Espoo, Finland).

### Real-time Quantitative RT-PCR Analyses of HCC Cell Lines

In cell lines and tissues, the relative expression ratio (R) of *SLIT-ROBO *and *AFP *transcripts (target gene) was measured based on a modified ΔΔCt formula [28] and normalized to *GAPDH *or *ACTB *(reference gene). In $\text{R}={\left({\text{E}}_{target}\right)}^{\Delta {\text{Ct}}_{target}(\text{control-sample})}/{\left({\text{E}}_{ref}\right)}^{\Delta {\text{Ct}}_{ref}(\text{control-sample})}$ formula, E_*target *_and E_*ref*_reflect PCR efficiencies of the primers for target genes and reference genes, respectively. PCR efficiency values for each primer pair was obtained by constructing a standard curve using threshold cycle (Ct) values derived from 6 data points, corresponding to 2-fold decrements of an original cDNA stock (duplicates were prepared for each dilution). The slope of the resulting curve was used to calculate the E value of primer pairs according to E = 2^-1/slope ^formula. PCR efficiencies of the genes ranged between 1.9 and 2.0. ΔCt was the difference between the Ct values of controls and samples.

In cell lines, *GAPDH *was the reference gene. ΔCt values were obtained by subtracting Ct values of individual genes (sample) from the average Ct value of all cell lines for that gene (control). All reactions were performed in duplicates and repeated at least twice using different batches of RNA preparations. Relative expression tables were established by representing ΔΔCt values in log2 base, and in all subsequent analyses these values were used.

Quantitative expression analyses were performed using DyNAmo™ HS SYBR^® ^Green qPCR Kit (Finnzymes, Espoo, Finland) on an iCycler iQ real-time PCR detection system (Bio-Rad, Richmond, CA). The PCR reaction was set according to the manufacturer's recommendations. Briefly for 1× reaction; 10 μl of 2× SYBR Green PCR Master Mix, 10 μM forward and reverse primers, and 1 μl of template cDNA were mixed in a total volume of 20 μl. After an initial 15 minutes of denaturation at 95°C, thermal cycling was performed at 94°C for 30 sec, 60–62°C for 30 sec (optimized for each primer pair), 72°C for 30 sec for a total of 50 cycles and a final extension step at 72°C for 10 min. In order to validate the production of a single target-specific PCR product, the amplification was followed by a melt curve protocol with an initial step at 55°C for 30 sec and 80 repeats of 0.5°C increments with 15 sec dwell time, from 55°C to 95°C.

### Real-time Quantitative RT-PCR Analyses of HCC Tissues

The expression of *SLIT-ROBO *and *AFP *genes in HCC was analyzed using a 96-well plate format TissueScan Liver Cancer Tissue qPCR Array 1 (Origene Technologies, Rockville, MD), which contained tissue cDNAs normalized against beta-actin. Real-time PCR protocol was applied as described by the manufacturer. Briefly, 30 μl of reaction mix containing 15 μl 2× SYBR Green PCR Master Mix and 10 μM forward and reverse primers was directly added to PCR-plate wells. Plate was placed on ice for 15 min for cDNAs to dissolve, and thermal cycling was performed according to above mentioned protocol. For each gene, mean Ct value of the normal tissue cDNAs was set as the control group and relative quantitative expression values were calculated with the ΔΔCt formula and were represented in log2 base by taking the *ACTB *Ct values as reference.

### Statistical Analysis

Using one-way ANOVA in R, mean expression levels of each gene were compared between high and low *AFP *expressing groups of HCC cell lines; and also between normal and tumor tissues of liver with respect to differentiation or stage [29]. Pairwise comparisons were made using Fisher's multiple pairwise comparison method in Minitab^® ^13.20 Statistical Software (Minitab Inc. 2000). Furthermore, two-way hierarchical cluster analysis was used to group cell lines and liver tissues with respect to the *SLIT-ROBO *expression patterns using Cluster and TreeView [30]. Pairwise correlations between *SLIT-ROBO *gene expression levels were calculated using Pearson's correlation coefficient. Moreover, a Mantel's association test was applied to compare cell line and tissue correlation matrices [29].

## Results

### QRT-PCR analyses reveal an association between *SLIT-ROBO *and *AFP *expression in HCC cell lines

We first investigated by RT-PCR the presence of *SLIT-ROBO *transcripts in 13 HCC and 1 hepatoblastoma cell lines. All genes were expressed at levels varying from none to strong after 40 cycles of amplification (Additional file 2). Since HCC cell lines were previously reported to cluster in two main molecular subtypes in terms of their *alpha-fetoprotein (AFP) *expression [31] we quantified *SLIT-ROBO *gene expression along with *AFP *transcript levels.

**Figure 1:**
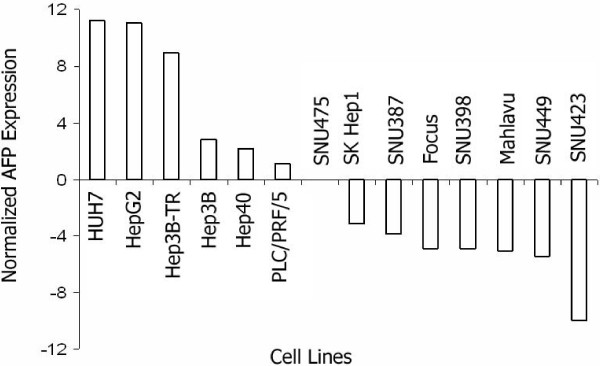
**Quantification of *AFP *transcript levels reveals two groups in HCC cell lines**. *GAPDH *normalized *AFP *expression was quantified by real-time RT-PCR and expression values of individual cell lines are calculated relative to the Ct average of all cell lines, and represented in log2 base. Samples were run in duplicates and the data are representative of two independent experiments.

In our first set of qRT-PCR experiments, cell lines with fibroblastoid morphology, including Focus, Mahlavu, SK Hep1, SNU387, SNU398, SNU423, SNU449 and SNU475, displayed low-*AFP *expression, while *AFP *was found to be overexpressed in epitheloid Hep40, Hep3B, Hep3B-TR, HepG2, HUH7 and PLC/PRF/5 cells (Figure 1). Second, we quantified *SLIT-ROBO *transcripts. *ROBO2 *and *SLIT1 *expression were up-regulated in high-*AFP *group of cells, except HepG2, which underexpressed *ROBO2 *(Additional file 3). On the other hand, both genes were found to be overexpressed in SNU398, one of the low-*AFP *group of cell lines. *SLIT3 *transcript levels sharply contrasted those of *ROBO2 *and *SLIT1*. Except SNU398, increased *SLIT3 *expression was found among low-*AFP *expressing group of cell lines, and also in high-*AFP *expressing Hep40 cell line. Interestingly, widespread expression of *ROBO1 *transcripts that we observed in RT-PCR turned out to be enhanced in cell lines with high-*AFP *background (Additional file 2 and 3). *ROBO4 *and *SLIT2 *transcript levels remained variable among HCC cell lines and no phenotype-based association could be observed for these genes. Our attempts to calculate PCR efficiency with two different *ROBO3 *primer pairs failed, and we discontinued qRT-PCR analysis of this gene.

A significant association of gene expression was found among *SLIT-ROBO *family genes and *AFP *based on Pearson's pairwise correlation coefficients (Table 1). In particular, expressions of *ROBO1 *and *SLIT3 *were significantly correlated with that of *AFP*, in a positive and negative manner, respectively.

**Table 1:** Genewise correlation of *SLIT-ROBO *and *AFP *genes in HCC cell lines

Gene	*ROBO1*	*ROBO2*	*ROBO4*	*SLIT1*	*SLIT2*	*SLIT3*	*AFP*
*ROBO1*	1						

*ROBO2*	0.6060*	1					

*ROBO4*	-0.2721	-0.2653	1				

*SLIT1*	0.4027	0.7623*	-0.3288	1			

*SLIT2*	0.1241	0.1891	0.5143	-0.0039	1		

*SLIT3*	-0.5698*	-0.6072*	0.3681	-0.5451*	-0.1003	1	

*AFP*	0.6533*	0.5108	-0.3016	0.4579	0.3544	-0.6605*	1

*: p < 0.05

### Cluster analyses identify two subgroups in HCC cell lines with respect to *SLIT-ROBO *expression levels

**Figure 2:**
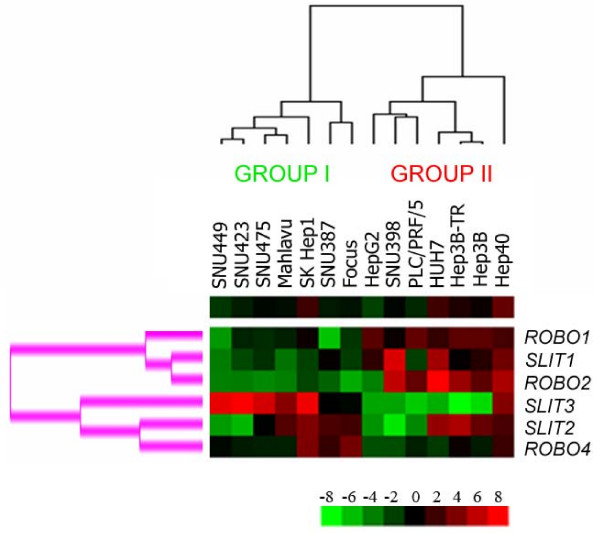
***SLIT-ROBO *expression establishes two groups, which predict *AFP *expression levels in HCC cell lines**. The means of normalized expression values of individual *SLIT-ROBO *genes in each cell line were used to establish the hierarchical clustering in Cluster program, and the results were visualized as a heatmap by TreeView. Overexpression and underexpression of individual genes relative to Ct average of all cell lines are represented by *red *and *green *colors, respectively. Upper single-row heatmap displays the average expression of all six genes across all cell lines. The tree at the top of the heatmap represents samplewise clustering. Based on their *AFP *expression levels, HCC cell lines are represented in two main subgroups indicated as Group I (low-*AFP*) and Group II (high-*AFP*) cells. The tree on the left of the heatmap (*pink*) represents genewise clustering.

Hierarchical clustering was performed in order to understand whether *SLIT-ROBO *expression predicts *AFP*-dependent grouping of HCC cell lines. In fact, in terms of *SLIT-ROBO *expression, low-*AFP *expressing SNU449, SNU423, SNU475, Mahlavu, SK Hep1, SNU387, and Focus cells grouped together (Group I), while high-*AFP *expressing HepG2, PLC/PRF/5, HUH7, Hep3B-TR, HEP-3B and Hep40 cell lines (Group II) clustered separately from the first group (Figure 2). Exceptionally, SNU398 cell line displayed a *SLIT-ROBO *expression pattern concordant with Group II despite its low-*AFP *background. The expression of *AFP *significantly differed between the two groups (p = 0.0001): Mean expression of *AFP *in Group I and Group II cells was -4.65 ± 2.75 and 6.20 ± 4.68, respectively.

Hierarchical clustering analysis also revealed a genewise segregation of *SLIT-ROBO *genes. Coordinate expression was observed in two main clusters, one of which grouping together *ROBO1, ROBO2 *and *SLIT1*, and the other *ROBO4*, *SLIT2*, and *SLIT3*, respectively (Figure 2). We also performed one-way ANOVA to identify individual *SLIT-ROBO *genes, which significantly separate HCC cell lines with high- and low-*AFP *expression. We validated that *ROBO1 *and *ROBO2 *expression were up-regulated in high-*AFP *group (p = 6.7 × 10^-4 ^and p = 0.013, respectively). Mean expression of *ROBO1 *in Group I was -1.73 ± 2.15 while in Group II cells increased to 2.37 ± 0.48. Similarly, *ROBO2 *mean expression values were found as -2.37 ± 3.31 and 3.11 ± 3.73, for Group I and Group II, respectively. In sharp contrast, *SLIT3 *was overexpressed in cell lines with low-*AFP *profile (Mean_GroupI _= 3.28 ± 4.43, Mean_GroupII _= -4.24 ± 4.06; p = 6.9 × 10^-3^).

### *SLIT-ROBO *family expression profile in liver tissues correlates with that in HCC cell lines

Next, we addressed the question of whether our cell line data translate to *in vivo *conditions. To this end, we quantified the expression of *SLIT-ROBO *and *AFP *genes using cDNAs of 8 tumor-adjacent normal and 35 tumor tissues (Additional file 4). Accordingly, only *ROBO1 *was found to be significantly overexpressed in tumor tissues (Mean_Normal _= 0 ± 0.63, Mean_Tumor _= 1.57 ± 1.63; p = 0.011). We also observed a down-regulated *ROBO4 *expression in tumor tissues with a p-value near significance (Mean_Normal _= 0 ± 1.03, Mean_Tumor _= -0.89 ± 1.30; p = 0.079). *AFP *displayed a highly variable expression, yet it was overexpressed in more than half of the tumors (18/35) (Additional file 4).

**Figure 3:**
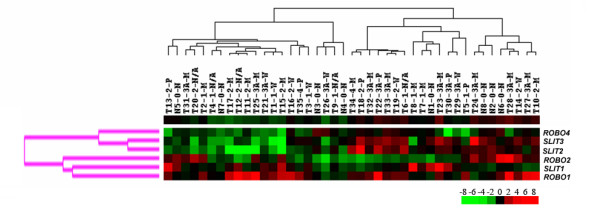
**Genewise clustering of *SLIT-ROBO *genes in HCC cell lines translates into liver tissues**. The normalized expression values of individual *SLIT-ROBO *genes in liver tissue samples (n = 43) were used to establish the hierarchical clustering in Cluster program and the results were visualized as a heatmap by TreeView. Overexpression and underexpression of individual genes relative to Ct average of normal tissues (n = 8) are represented by *red *and *green *colors, respectively. Upper single-row heatmap displays the average expression of all six genes across all tissues. The tree at the top of the heatmap represents samplewise clustering, and the tree on the left of the heatmap (*pink*) represents genewise clustering. T1-35: tumors; N1-8: normal tissues; 0: stage 0 (non-tumor); 1, 2, 3A, 4: stage 1, stage 2, stage 3A, stage 4 HCC, respectively; N: tumor-adjacent normal tissue; W: well-differentiated; M: moderately differentiated; P: poorly differentiated; N/A: not-assigned.

Hierarchical clustering grouped *SLIT-ROBO *genes in a similar manner as in cell lines. *ROBO1*, *ROBO2 *and *SLIT1 *clustered together, whereas *ROBO4*, *SLIT2 *and *SLIT3 *formed another cluster (Figure 3). Two main clusters appeared among tissue samples; however, no significant association of clusters was found with differentiation state or staging of tumors (Wilcoxon rank sum test) and *AFP *expression (one-way ANOVA).

Expression correlation amongst individual *SLIT-ROBO *and *AFP *genes in liver tissues was analyzed by Pearson's correlation analysis in R (Table 2). Accordingly, expression of *ROBO1 *in HCC samples was positively correlated with that of *AFP *whereas *ROBO4 *and *AFP *were inversely correlated in terms of their expression. Mantel's permutation test, performed to compare gene expression correlation matrices of cell lines and tissues (described in Table 1 and Table 2, respectively), indicated that gene-to-gene correlation patterns in both sample groups were significantly associated (r = 0.49; p < 0.02).

**Table 2:** Genewise correlation of *SLIT-ROBO *and *AFP *genes in liver tissues

Gene	*ROBO1*	*ROBO2*	*ROBO4*	*SLIT1*	*SLIT2*	*SLIT3*	*AFP*
*ROBO1*	1						

*ROBO2*	0.1835	1					

*ROBO4*	-0.1879	-0.2134	1				

*SLIT1*	0.0298	0.002	-0.3793*	1			

*SLIT2*	-0.3679*	-0.0078	0.3432*	-0.1389	1		

*SLIT3*	-0.0973	-0.0027	0.2596	-0.0965	0.6844***	1	

*AFP*	0.3679*	0.2324	-0.3699*	0.0924	-0.0769	-0.1638	1

***: p < 0.001; *: p < 0.05

### *ROBO1 *expression differentiates normal tissues from tumors with respect to both stage and differentiation status

**Figure 4:**
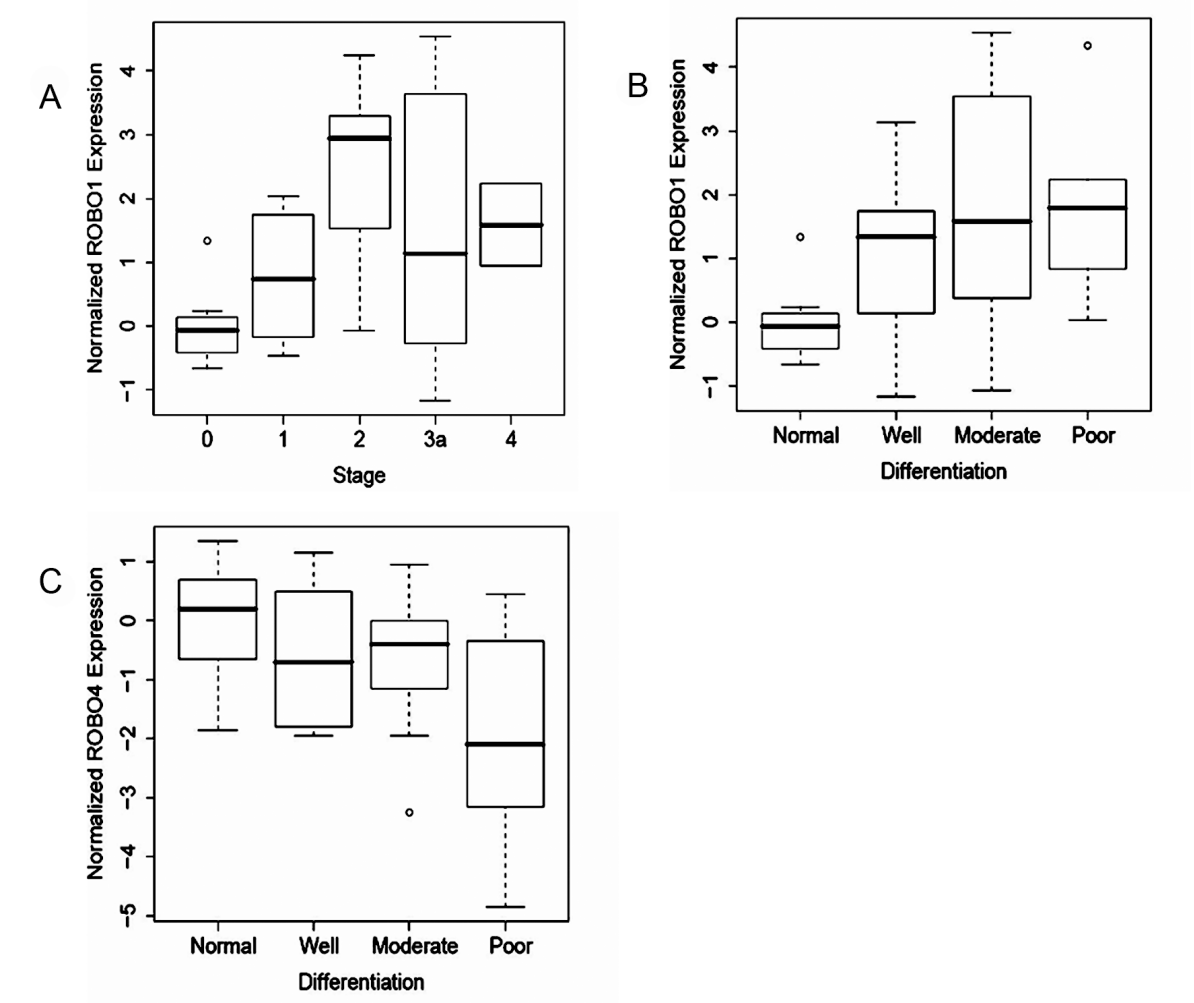
***ROBO1 *and *ROBO4 *expression discriminate between different stage and differentiation groups of liver tissues**. Expression values of *SLIT-ROBO *genes were compared using one-way ANOVA in liver tissue samples that are stratified according to their stages and differentiation status and represented as boxplots. (A) *ROBO1 *significantly differentiates one staging group from the others (p = 0.018). (B) The expression of *ROBO1 *also discriminates between different differentiation status of liver tissues (p = 0.031). (C) *ROBO4 *also significantly separates one differentiation group among all (p = 0.039).

We also performed one-way ANOVA followed by Fisher's multiple pairwise comparisons in order to explore whether gene expression characteristics of *SLIT-ROBO *family members discriminate among liver tissues with respect to differentiation status and staging of the samples. The tissue samples lacking stage or differentiation information in the pathological reports were left out of analysis. With these criteria, a total of 43 liver tissue samples for stage and of 38 tissue samples for differentiation status were analyzed. According to one-way ANOVA, *ROBO1 *differentiated liver tissue samples on both the stage (p = 0.018) and differentiation status (p = 0.031) dependent manner (Figure 4A, B). *ROBO4 *expression significantly discriminated tissues only with respect to their differentiation status (p = 0.039) (Figure 4C). Moreover, Fisher's pairwise comparison analyses revealed that *ROBO1*, *SLIT2 *and *ROBO4 *significantly discriminated between different histopathological subgroups both in terms of differentiation status and/or tumor staging (Table 3).

**Table 3:** Differentially expressed *SLIT-ROBO *genes between histopathological subgroups of liver tissues

**Stage**	***n***	**Mean expression (SE)**	Differentially expressed gene*
0	8	0 (0.22)	*ROBO1*
2	11	2.37 (0.41)	

0	8	0 (0.22)	*ROBO1*
3	13	1.43 (0.57)	

1	9	0.79 (0.33)	*ROBO1*
2	11	2.37 (0.41)	

2	11	-2.29 (1.15)	*SLIT2*
3	13	-0.96 (0.44)	

**Differentiation state**	***n***	**Mean expression (SE)**	Differentially expressed gene*

Normal	8	0 (0.22)	*ROBO1*
Moderate	16	1.89 (0.44)	

Normal	8	0 (0.22)	*ROBO1*
Poor	6	1.84 (0.60)	

Normal	8	0 (0.36)	*ROBO4*
Poor	6	-2.02 (0.80)	

Well	8	-0.60 (0.43)	*ROBO4*
Poor	6	-2.02 (0.80)	

Moderate	16	-0.66 (0.25)	*ROBO4*
Poor	6	-2.02 (0.80)	

Moderate	16	-1.28 (0.78)	*SLIT2*
Poor	6	1.01 (0.88)	

* Pairs of stage and differentiation subgroups were compared in Fisher's pairwise comparison analysis and only the genes that significantly discriminate between subgroups were represented (p < 0.05). SE: standard error.

## Discussion

HCC remains the fifth most common cancer worldwide and is at the third rank in cancer-caused deaths. The prognosis of patients is generally very poor with a 5-year relative survival of only 7% [32]. The elucidation of molecular mechanisms governing hepatocarcinogenesis is therefore of high priority not only for the better understanding of the disease, but also to develop more effective therapies. To achieve this goal, functional genomics studies could provide valuable information with regard to genes differentially expressed between HCC and normal liver. A collective analysis of expression signature of *SLIT-ROBO *family genes has not been assessed yet in liver tumor. Here, we showed the co-regulation of *SLIT-ROBO *genes in HCC. In both the HCC cell lines and liver tissues, *ROBO1*, *ROBO2*, *SLIT1*, and *ROBO4*, *SLIT2*, *SLIT3 *showed coordinate expression as two distinct modules, yet displaying high variability at gene level within each module. Additionally, *SLIT-ROBO *expression was able to predict *AFP *status of HCC cell lines, and thereby establishing two groups with low- and high-*AFP *expressions.

Except *ROBO3*, all genes were found to be expressed at different levels in our analyses. A preferential up- and down-regulation of *SLIT-ROBO *genes occurred depending on the *AFP *expression status of HCC cell lines. *ROBO1*, *ROBO2 *and to a lesser extent *SLIT1 *were overexpressed, whereas *SLIT3 *was underexpressed in high-*AFP *group. *ROBO4 *also tended to be down-regulated in this group. However, *SLIT2 *was expressed in most of the cell lines, regardless of the *AFP *expression status.

We also quantified *SLIT-ROBO *expression in 8 tumor-adjacent normal liver tissues and 35 HCC tumors. We found that genewise clustering observed in HCC cell lines were conserved in tissues: *ROBO1*, *ROBO2*, *SLIT1 *and *ROBO4*, *SLIT2*, *SLIT3 *were coordinately expressed, respectively. We also noticed two main subgroups in tissue samples but the observed *AFP *dependent subgrouping in HCC cell lines did not translate into the tissue analysis, except that *AFP *and *ROBO1 *expression was significantly correlated in both HCC cell lines and tissues. This discrepancy might be partly due to the heterogeneity of tissues. HCC cell lines were more homogenous when compared to tissue samples, which may contain stromal cells, endothelial cells, immune cells or any other tumor infiltrating cells. Moreover, our normal liver samples were tumor-adjacent tissues, which may harbor genetic changes of tumor microenvironment, and therefore may not reveal the actual molecular characteristics of a tumor-free normal liver.

*ROBO1 *transcript was present in all cell lines that were examined and it was significantly up-regulated in the analyzed HCC tissues, in which its overexpression culminated in later stages and as tumors progress to a less differentiated state. These data were in agreement with a recent report that demonstrated ROBO1 as an HCC antigen and proposing it as both a diagnostic marker and therapeutic target for HCC [19]. *SLIT2 *was present in most of the tumor tissues and HCC cell lines although at variable levels. Such variability might explain the clustering of *SLIT2 *in a different group than *ROBO1 *and *ROBO2*, yet it is likely to be the main ligand for ROBO receptors. Nevertheless, this does not exclude interactions between other SLIT and ROBO members, nor it does the possible ligand-independent activities of ROBO receptors in HCC. Additionally, *SLIT2 *and *ROBO1 *were both upregulated in HCCs with advanced stages and poor differentiation status (Figure 3 and Table 3). These findings also are in agreement with the expression of SLITs specifically in poorly differentiated HCCs [19]. Furthermore, in a tumor xenograft model, SLIT2-ROBO1 signaling was shown to have a role in angiogenesis, which supports tumor growth and metastasis [13].

Although *ROBO4 *was shown to be specific to vasculature [11], we observed varying levels of *ROBO4 *transcript in HCC cell lines. The *ROBO4 *transcripts in these cells might be partly explained by the presence of side population cells with stem cell characteristics that express markers of the vascular endothelium [33]. One may also consider a possible regulation of *ROBO4 *expression in liver tumors. In tissue expression analyses, we found that *ROBO4 *expression is significantly down-regulated in poorly differentiated tumors, indicating that ROBO4 function is not essential for the maintenance of tumor at this step of hepatocarcinogenesis. In fact, recent findings indicated that SLIT2-ROBO4 interactions inhibited angiogenesis [34].

It is very likely that *SLIT2-ROBO1-ROBO4 *might contribute to some of the variability associated with the differentiation status of HCC while expression of *ROBO1 *and *SLIT2 *also helps explain the stage differences in this cancer. However, the expression variability is high among liver tumors suggesting that a combinatorial code with a possibility of ligand redundancy might be at work in hepotocellular carcinoma, which prompts further functional studies that include knock-down and overexpression.

A global gene expression analysis by microarray technology in 19 HCC cell lines revealed two molecular subtypes depending on their *AFP *expression level [31]. Of 14 HCC cell lines that we studied, 13 were included in that study and the *SLIT-ROBO *dependent subgrouping in our analysis was parallel to the *AFP *subgrouping previously observed, verifying the reliability of our cell line panel. *ROBO1 *and *SLIT3 *were the genes that were most significantly correlated with *AFP *expression in a positive and negative manner, respectively. Genes regulating extracellular matrix establishment or remodeling and cell adhesion were shown to be differentially expressed between the HCC cell line subgroups [31]. Cells that were defined to be more metastatic and motile correspond to cell lines that cluster as Group I in our study. Given the important roles of SLIT-ROBO associated signaling molecules like ENA, ABL, and several GTPase activating proteins in cytoskeletal reorganization and cell motility [35,36], the connection between SLIT-ROBO signaling and HCC tumor cell invasion and metastasis remains to be further described.

## Conclusion

Here, we defined the overexpression of *ROBO1 *and the variable expression of other *SLIT-ROBO *family members in HCC. Especially, downregulation of *ROBO4 *and upregulation of *SLIT2 *mark late stage and poorly differentiated HCCs. We have also shown that the collective expression of these genes occurs in a coordinated fashion in two main groups suggesting that SLIT-ROBO signaling is modular in nature, and that each module shows intrinsic variability. Our results help increase our understanding of pathological expression pattern of *SLIT-ROBO *family in HCC with potential for diagnostic applications. Elucidation of the mechanisms acting on the transcriptional regulation of SLIT-ROBO signaling pathway, such as alternative splicing, copy number variability and ligand/receptor redundancy in both HCC and other pathophysiological contexts will contribute to a better understanding of hepatocarcinogenesis.

## Abbreviations

HCC: hepatocellular carcinoma; *ROBO*: roundabout; *AFP*: alpha-fetoprotein; *GAPDH*: glyceraldehyde-3-phosphate dehydrogenase; *ACTB*: beta-actin.

## Competing interests

The authors declare that they have no competing interests.

## Authors' contributions

MEA designed the primers, performed RT-PCR and real-time PCR experiments, and contributed to statistical analyses. OK participated in the design of the study, supervised the statistical analyses, and helped draft the manuscript. TY designed and coordinated the study, and finalized the manuscript. All authors read and approved the final manuscript.

## Pre-publication history

The pre-publication history for this paper can be accessed here:



## Supplementary Material

Additional file 1 **Clinicopathological characteristics of normal liver and HCC samples.** This table gives Diagnosis/Histology, stage and differentiation information for normal and tumor tissues used in the study. Click here for file

Additional file 2 ***SLIT-ROBO *****genes are expressed in HCC cell lines at varying levels.** Agarose gel electrophoresis image of *SLIT-ROBO *transcripts in 14 HCC cell lines after 40 cycles of RT-PCR amplification. Click here for file

Additional file 3 **Relative expression of *SLIT-ROBO *and *AFP *genes in HCC cell lines.** This table lists the ΔΔCt values in log2 base for *SLIT-ROBO *and *AFP *transcripts in 14 HCC cell lines used in the study. Click here for file

Additional file 4 **Relative expression of *SLIT-ROBO *and *AFP *genes in normal liver and HCC samples.** This table lists the ΔΔCt values in log2 base for *SLIT-ROBO *and *AFP *transcripts in 8 normal liver and 35 HCC samples used in the study. Click here for file
