# Supplementary material for: Quantification of SLIT-ROBO transcripts in hepatocellular carcinoma reveals two groups of genes with coordinate expression
Source: BMC Cancer. 2008 Dec 29;8:392. doi: 10.1186/1471-2407-8-392 (PMC2632672; doi:10.1186/1471-2407-8-392)
Supplement: Additional file 1 — Clinicopathological characteristics of normal liver and HCC samples. This table gives Diagnosis/Histology, stage and differentiation information for normal and tumor tissues used in the study. [file 1471-2407-8-392-S1.pdf]

Additional file 1. Clinicopathological characteristics of normal liver and HCC samples

Sample #	***Appearance***	***Diagnosis/Histology***	***Differentiation***	***Stage***
**N1**	Normal	hcc	tumor-adjacent normal	0
**N2**	Normal	hcc/adenocarcinoma	tumor-adjacent normal	0
**N3**	Normal	hcc	tumor-adjacent normal	0
**N4**	Normal	hcc	tumor-adjacent normal	0
**N5**	Normal	granuloma	tumor-adjacent normal	0
**N6**	Normal	nodular hyperplasia of liver, focal	tumor-adjacent normal	0
**N7**	Normal	hcc	tumor-adjacent normal	0
**N8**	Normal	hcc	tumor-adjacent normal	0
**T1**	Tumor	hcc	well	1
**T2**	Tumor	hcc	moderate	1
**T3**	Tumor	hcc	well	1
**T4**	Tumor	hcc	N/A	1
**T5**	Tumor	hcc	poor	1
**T6**	Tumor	hcc	N/A	1
**T7**	Tumor	hcc	moderate	1
**T8**	Tumor	hcc	moderate	1
**T9**	Tumor	hcc	N/A	1
**T10**	Tumor	hcc	moderate	2
**T11**	Tumor	hcc/adenocarcinoma	moderate	2
**T12**	Tumor	hcc/invasive	N/A	2
**T13**	Tumor	hcc/adenocarcinoma	poor	2
**T14**	Tumor	hcc	well	2
**T15**	Tumor	hcc	moderate	2
**T16**	Tumor	hcc	well	2
**T17**	Tumor	hcc	moderate	2
**T18**	Tumor	hcc/adenocarcinoma	poor	2
**T19**	Tumor	hcc	well	2
**T20**	Tumor	hcc/invasive	N/A	2
**T21**	Tumor	hcc	well	3a
**T22**	Tumor	hcc	poor	3a
**T23**	Tumor	hcc	moderate	3a
**T24**	Tumor	hcc	moderate	3a
**T25**	Tumor	hcc	moderate	3a
**T26**	Tumor	hcc	well	3a
**T27**	Tumor	hcc	moderate	3a
**T28**	Tumor	hcc	moderate	3a
**T29**	Tumor	hcc	well	3a
**T30**	Tumor	hcc	poor	3a
**T31**	Tumor	hcc	moderate	3a
**T32**	Tumor	hcc	moderate	3a
**T33**	Tumor	hcc	moderate	3a
**T34**	Tumor	hcc	moderate	4
**T35**	Tumor	hcc/from omentum	poor	4
