# Supplementary figures and images for: Quantification of SLIT-ROBO transcripts in hepatocellular carcinoma reveals two groups of genes with coordinate expression
Source: BMC Cancer. 2008 Dec 29;8:392. doi: 10.1186/1471-2407-8-392 (PMC2632672; doi:10.1186/1471-2407-8-392)

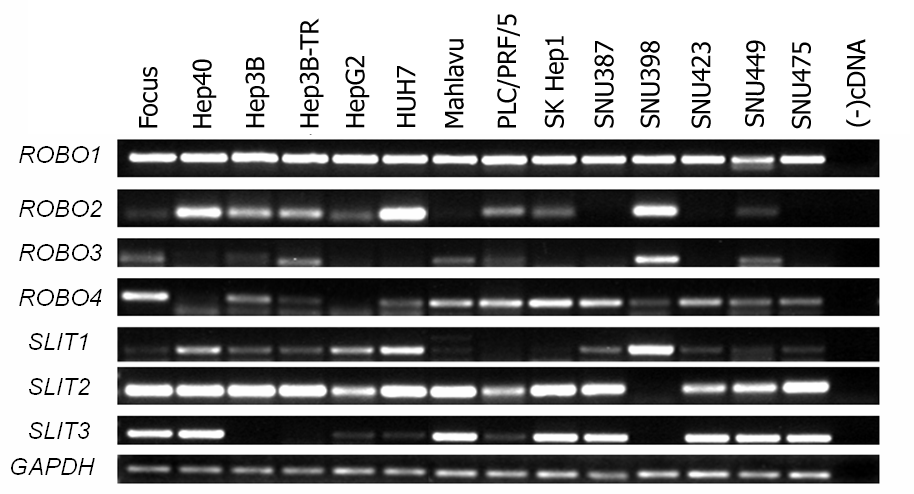

Supplement: Additional file 2 — SLIT-ROBO genes are expressed in HCC cell lines at varying levels. Agarose gel electrophoresis image of SLIT-ROBO transcripts in 14 HCC cell lines after 40 cycles of RT-PCR amplification. [file 1471-2407-8-392-S2.tiff]
