# Supplementary material for: Quantification of SLIT-ROBO transcripts in hepatocellular carcinoma reveals two groups of genes with coordinate expression
Source: BMC Cancer. 2008 Dec 29;8:392. doi: 10.1186/1471-2407-8-392 (PMC2632672; doi:10.1186/1471-2407-8-392)
Supplement: Additional file 3 — Relative expression of SLIT-ROBO and AFP genes in HCC cell lines. This table lists the ΔΔCt values in log2 base for SLIT-ROBO and AFP transcripts in 14 HCC cell lines used in the study. [file 1471-2407-8-392-S3.pdf]

Additional file 3. Relative expression of *SLIT-ROBO* and *AFP* genes in HCC cell lines

	ROBO1	*ROBO2*	*ROBO4*	*SLIT1*	*SLIT2*	*SLIT3*	*AFP*
Focus	-1.53	-4.95	3.32	-1.72	1.85	-0.22	-4.86
Hep40	2.05	4.88	1.67	3.23	2.08	3.83	2.19
Hep3B	2.62	2.66	-0.78	1.10	2.88	-6.32	2.78
Hep3B-TR	2.67	4.46	0.40	0.23	4.95	-7.35	8.94
HepG2	2.52	-3.65	-2.27	1.35	-4.22	-4.77	11.03
Huh7	1.55	7.44	-1.62	4.60	4.25	-5.00	11.19
Mahlavu	-0.93	-3.61	-0.87	-3.30	2.33	3.90	-5.07
PLC	2.80	2.85	-0.89	-1.75	-3.95	-5.82	1.09
SKHep1	0.45	-1.99	3.58	-1.87	3.68	6.93	-3.14
Snu387	-5.58	-2.83	1.56	0.23	2.15	0.03	-3.86
Snu398	0.35	5.54	-2.18	6.28	-6.65	-4.67	-4.87
Snu423	-1.03	-3.56	-0.79	-2.05	-5.25	7.65	-9.94
Snu449	-4.38	-3.65	-0.23	-3.57	-4.40	6.63	-5.43
Snu475	-1.23	-3.93	-0.88	-1.37	-0.10	6.03	-0.04
