# Supplementary material for: Quantification of SLIT-ROBO transcripts in hepatocellular carcinoma reveals two groups of genes with coordinate expression
Source: BMC Cancer. 2008 Dec 29;8:392. doi: 10.1186/1471-2407-8-392 (PMC2632672; doi:10.1186/1471-2407-8-392)
Supplement: Additional file 4 — Relative expression of SLIT-ROBO and AFP genes in normal liver and HCC samples. This table lists the ΔΔCt values in log2 base for SLIT-ROBO and AFP transcripts in 8 normal liver and 35 HCC samples used in the study. [file 1471-2407-8-392-S4.pdf]

**Additional file 4**. Relative expression of *SLIT-ROBO* and *AFP* genes in normal liver and HCC samples.

| ***Sample #*** | ***ROBO1*** | ***ROBO2*** | ***ROBO4*** | ***SLIT1*** | ***SLIT2*** | ***SLIT3*** | ***AFP*** |
|---|---|---|---|---|---|---|---|
| **N1** | -0.66 | -2.49 | 0.15 | 2.76 | 0.03 | 1.09 | 2.06 |
| **N2** | 0.24 | 1.61 | 0.35 | -1.04 | 1.63 | 1.09 | 1.98 |
| **N3** | 0.04 | -2.39 | 1.35 | -0.94 | 0.43 | -0.21 | 1.23 |
| **N4** | -0.66 | -2.49 | -0.75 | -1.04 | -1.88 | 0.09 | -0.74 |
| **N5** | -0.06 | 1.81 | -0.55 | 1.76 | -0.78 | -1.21 | -0.96 |
| **N6** | 1.34 | 3.71 | 1.05 | -0.14 | 0.83 | -0.21 | -1.45 |
| **N7** | -0.06 | -0.69 | -1.85 | -0.84 | -1.18 | -2.41 | -1.30 |
| **N8** | -0.16 | 0.91 | 0.25 | -0.54 | 0.93 | 1.79 | -0.83 |
| **T1** | 1.84 | -0.79 | -1.75 | 0.66 | -2.68 | -3.81 | 5.46 |
| **T2** | 1.74 | 1.71 | -0.65 | -0.34 | -1.48 | -0.71 | -2.24 |
| **T3** | 0.74 | -0.69 | -1.05 | 0.76 | -0.58 | 0.39 | 0.13 |
| **T4** | 0.14 | -1.59 | -1.05 | -0.14 | -2.68 | -2.41 | 0.08 |
| **T5** | 2.04 | 0.51 | -3.15 | -0.34 | 0.83 | -1.11 | 5.48 |
| **T6** | 1.44 | -0.99 | 0.25 | 0.46 | 2.63 | 1.89 | 0.00 |
| **T7** | -0.16 | -1.49 | -0.15 | 0.86 | 0.33 | -0.11 | -1.01 |
| **T8** | -0.16 | -1.49 | 0.15 | 4.56 | -0.38 | -2.81 | -1.77 |
| **T9** | -0.46 | -1.39 | 0.15 | 0.06 | 0.43 | -0.71 | -0.99 |
| **T10** | 3.84 | 0.91 | -0.25 | -1.04 | 0.73 | 1.59 | 8.68 |
| **T11** | 3.44 | -2.09 | -0.35 | -0.64 | -4.98 | -0.91 | 4.75 |
| **T12** | 4.24 | -2.69 | -1.95 | 1.76 | -7.68 | -0.71 | -0.09 |
| **T13** | 1.54 | 1.01 | -2.75 | 3.56 | -1.68 | -0.51 | 6.72 |
| **T14** | 1.64 | 2.41 | -0.35 | -0.54 | 0.63 | 1.59 | -0.07 |
| **T15** | 3.04 | 1.61 | -3.25 | 2.76 | -5.38 | -4.11 | 11.63 |
| **T16** | 3.14 | -0.99 | 0.35 | 0.46 | -1.28 | -0.01 | 5.09 |
| **T17** | 2.94 | -0.99 | -1.25 | 0.46 | -7.88 | -2.81 | -6.19 |
| **T18** | 0.84 | -2.89 | 0.45 | -1.44 | 3.93 | 2.49 | -1.81 |
| **T19** | 1.54 | -1.59 | 0.65 | -0.14 | 1.03 | 1.59 | 3.13 |
| **T20** | -0.06 | 2.61 | -0.95 | 0.06 | -2.68 | -3.01 | 0.87 |
| **T21** | 1.14 | -0.29 | -1.95 | 1.16 | -0.98 | -1.91 | 4.09 |
| **T22** | 4.34 | -1.89 | -0.35 | -0.44 | 2.83 | 1.79 | -0.73 |
| **T23** | 1.44 | -0.79 | -1.05 | 5.96 | 1.03 | 2.59 | -6.07 |
| **T24** | -1.06 | 2.41 | -1.05 | -0.34 | 1.13 | 5.09 | -9.53 |
| **T25** | 4.54 | 1.41 | -1.55 | -0.24 | -6.38 | -3.11 | -4.18 |
| **T26** | -1.16 | -3.49 | 1.15 | -2.04 | -1.28 | -2.71 | -10.13 |
| **T27** | 3.64 | 0.71 | 0.25 | -1.24 | -0.78 | 0.69 | -3.99 |
| **T28** | 3.94 | 6.11 | -1.95 | -0.14 | 0.23 | 0.49 | 11.36 |
| **T29** | -0.46 | -0.99 | -1.85 | 0.46 | 0.13 | 0.59 | -0.17 |
| **T30** | 0.04 | -0.99 | -4.85 | 2.46 | 1.13 | 1.59 | 1.95 |
| **T31** | -0.26 | 1.11 | -0.45 | -0.44 | -1.08 | -0.31 | 2.41 |
| **T32** | 1.44 | -2.19 | 0.25 | -0.74 | 0.63 | 1.29 | 1.94 |
| **T33** | 1.04 | -1.29 | 0.95 | 0.16 | 1.43 | 2.29 | -2.67 |
| **T34** | 0.94 | -2.19 | -0.25 | -0.74 | 2.33 | 0.39 | -5.27 |
| **T35** | 2.24 | -1.99 | -1.45 | -0.54 | -0.98 | 0.39 | 9.94 |
